# Managing disclosure outcomes in intelligence interviews

**DOI:** 10.1098/rsos.240635

**Published:** 2024-06-19

**Authors:** David A. Neequaye, Timothy J. Luke, Kristina Kollback

**Affiliations:** ^1^ Department of Psychology, University of Gothenburg, Gothenburg, Sweden; ^2^ Department of Psychology, Lancaster University, Lancaster, UK

**Keywords:** disclosure, intelligence interviewing, information management, self-interest dilemma

## Abstract

We introduce the disclosure-outcomes management model. The model views disclosure in intelligence interviews as a behaviour interviewees use to profitably navigate self-interest dilemmas. We theorized that interviewees compare the potential outcomes of disclosing to their self-interests. They evaluate the extent to which disclosure will facilitate or impede those self-interests: an interviewee’s self-interest dilemma elicits cooperation with respect to some information but not other information. A Preliminary Study (*N* = 300) supported the model’s predictions. We proposed a Replication Study (*N* = 369) to examine the model further. Participants assumed the role of an intelligence source undergoing an interview. They decided what information to disclose, contending the typical dilemma in an intelligence interview wherein disclosure could jeopardize or advance their self-interests. The results from the Preliminary and Replication studies were broadly in line with our proposition: perceived benefits positively influenced the likelihood of disclosing. However, a negative interaction between costs and benefits observed in the Preliminary Study did not replicate. That finding may be due to power constraints, not evidence against the existence of an interaction effect. Our proposal that—generally speaking—interviewees are likelier to disclose information units that seem less versus more risky requires further examination. Individual-level sensitivity to benefits, costs and their co-occurrence varied substantially in our studies. We discuss avenues for future research.

## 1. Managing disclosure outcomes in intelligence interviews

Intelligence interviews are social interactions in which a human collector of information—an interviewer—solicits such information from a human source—the interviewee [1]. An intelligence interview aims to obtain information relevant to national or international security, criminal activity or military operations [2]. Thus, gathering accurate and useful information from the interviewee is paramount [3]. The present work aims to propose and examine some mechanisms underlying information disclosure in human intelligence (HUMINT) interviews.

A close inspection of the existing literature reveals a considerable focus on interviewers. The majority of the research centres on developing interviewing approaches that improve the amount of information interviewers can elicit [4–6]. Researchers have focused less on examining the psychological mechanisms that drive interviewees’ disclosure. A better understanding of those mechanisms will enhance predictions about the range of influences questioning methods may exert. We offer a theoretical framework called the disclosure-outcomes management model.

In the present perspective, intelligence interviewees’ disclosure and non-disclosure of information are forms of decision-making, deciding *whether* to disclose. We view information disclosure decisions as a form of self-interest behaviour interviewees perform to achieve a preferred result. The term ‘self-interest’ broadly encompasses any outcome an interviewee may want to achieve or avoid. Those outcomes need not apply directly to the interviewee; they could act in the best interests of other associates.

We draw on subjective expected utility [7] to explain how interviewees decide what they could disclose. Accordingly, we propose that interviewees are likelier to disclose information they expect would yield positive utility versus negative utility, namely desirable rather than undesirable outcomes. This approach assumes that people consider the potential outcomes of their behaviours, and they typically enact behaviours intended to maximize personal benefits and incur the least costs [8,9]. This decision-making process does not necessarily correspond to the actual benefits and costs associated with a given decision. The perceived benefits and costs influence people’s decisions about what information they could disclose.

Past research on suspect interrogation supports our view. Suspects otherwise motivated to conceal information often volunteer details they believe their interviewer may already know [10–12]. That behaviour allows such suspects to appear cooperative and reap the corresponding benefits while avoiding the costs of appearing uncooperative. We argue that interviewees’ cooperativeness through disclosure can be conceptualized as a case of decision-making to achieve desired outcomes at minimal costs.

### 1.1. Navigating conflicting goals

Anecdotal evidence about intelligence interviews suggests that interviewees typically face a self-interest dilemma [13,14]. Interviewees often experience conflicting goals: some desired outcomes preclude other goals or vie for resources with other goals. A core tenet of the present model is this: *goals that can be accomplished by disclosing and withholding the same information are often in tension*. On the one hand, interviewees aim to offer some level of cooperation. That goal may or may not align with satisfying the interviewer’s request for information (or information objective). Importantly, the motive to cooperate usually comes with some material benefit that the interviewee considers advantageous. An example is sharing information that incriminates a narcotic peddling gang to help interdict them, thereby improving community safety. Conversely, interviewees aim to safeguard themselves from risks. Protecting oneself from the gang’s reprisal is an example of safeguarding oneself from risks. An interviewee could achieve such a motive by withholding or non-disclosure of information that incriminates the gang.

Intelligence interviewing researchers have attempted to incorporate goal conflict in their research designs. They typically induce some motivation to cooperate in their experiments [11,15]. Participants assuming the role of mock interviewees receive a twofold instruction on how to engage with a prospective interviewer. They receive instructions to be cooperative because assisting the interviewer is necessary for some benefit. The participants are also told to be resistant due to the risks of disclosure, for example, having strong ties to a mock terror group. The instruction ensures that participants have identical motivations before researchers examine an interviewing approach’s efficacy. Researchers often examine the effectiveness of inducing disclosure by testing an approach against the method of direct questioning.

The twofold instruction has proven useful; all the participants usually disclose some information. However, the participants do not disclose all of the information at their disposal. For example, Luke [11] reviewed the experimental research of a subtle information elicitation approach—the Scharff technique. Across the studies reviewed, the group means for information disclosure in the direct questioning conditions were always well above 0 [11]. On average, people interviewed with direct questioning regularly provide at least some of the information they possess. Complete withholding is the exception rather than the rule. Therefore, we can infer that interviewees presumably perceive some benefit in disclosing such information. Or, at least, that disclosure is not necessarily so opposed to their goals that they completely refuse to do it when directly prompted. Put simply, interviewees usually reveal some information when interviewers ask direct questions.

We propose that an interviewee considers how to navigate the conflicting goals of their self-interest dilemma in an interview. That evaluation determines what the interviewee decides to disclose to the interviewer. However, we do not construe such cooperativeness as a property of an interviewee *per se*. Rather, an interviewee’s cooperativeness in providing information varies within the individual; the variation occurs between different pieces of information the interviewee holds. That is to say, an interviewee may be likely to cooperate with respect to some information but not other information.

Here, the unit of analysis is, therefore, pieces of information nested within interviewees. We theorize that interviewees consider how revealing what they know might advance or jeopardize their self-interests. That consideration produces varying likelihoods of disclosing each piece of information rather than a global motivation. Interviewees *do not* have a motivation to cooperate that applies equally to all the information they hold. Instead, an interviewee will be more likely to disclose some pieces of information and less likely to disclose others. Unless stated otherwise, we use the term ‘disclosure’ in relation to a *specific piece of information* rather than the amount of information an interviewee shares. The interviewee possesses some number of information items, and the likelihood of disclosing each piece of information can vary.

The focus on decision-making concerning specific information items differentiates the present perspective from similar models. For instance, the interrogation decision-making model [16] also draws on expected utility to theorize about interviewees’ decision-making. Arguably, that model attends to broader level behaviours—decision-making on whether to confess or deny guilt. The disclosure-outcomes management model concentrates on behaviour at a lower level: how interviewees choose to reveal *specific* details, for example, the information a confession or denial contains. Moreover, the current model is not limited to culpability. The model elucidates the mechanisms of disclosure whenever interviewees contend with a self-interest dilemma. Those dilemmas do not always involve an interviewee’s potential guilt, for example, an ordinary citizen informing on a criminal gang.

Considering pieces of information as the primary unit of analysis offers many conceptual and analytic insights. By focusing on lower-level units, we can begin to offer idiographic predictions—hypotheses about the behaviour of specific individuals rather than groups of people. Additionally, we may consider how person-level variables (e.g. personality) might interact with the properties of information to influence that person’s likelihood of disclosing that information. Such considerations may lead to more sophisticated predictions about behaviour.

Some past data speak to the plausibility of our theorizing. An example is a study by Luke *et al.* [12] examining the disclosure of guilty and innocent mock suspects. The researchers informed the suspects that their interviewer might hold incriminating CCTV camera footage. The results indicated that the guilty suspects, as opposed to the innocent ones, behaved in two distinct ways. The guilty suspects tended to deny involvement entirely or they disclosed substantial amounts of accurate information about the activities in question (i. guilty knowledge) without admitting culpability.

The suspects did not know what exactly the footage might have contained. In that situation, disclosing the guilty knowledge could be beneficial by making the suspect appear cooperative and consistent with the evidence. Alternatively, the disclosure could severely damage the suspect’s claim of innocence. Here, the expected consequences of disclosure are both desirable and detrimental, respectively. Luke *et al.* [12] found that the distribution of information disclosures formed a bimodal distribution. This finding supports the view that self-interest dilemmas play a significant role in determining interviewees’ disclosure. Those who deemed appearing consistent with the evidence a worthwhile pursuit disclosed the guilty knowledge to advance that interest. Conversely, those who wanted to avoid incrimination, no matter what, refrained from disclosing the guilty knowledge. Other studies have produced results similar to the findings of Luke *et al.* [12] just described [10,17].

In developing that argument, we draw on the well-established notion that people typically prefer to achieve perceived benefits at minimal perceived costs. Disclosure or non-disclosure is an interviewee’s attempt to profitably navigate a self-interest dilemma. We propose that interviewees try to profitably navigate the network of conflicting goals by considering, at least intuitively, what information could be disclosed to advance their self-interests. Favourably navigating the self-interest dilemma is the interviewee’s overarching goal. That goal includes (i) sating the interviewer’s information objectives by sharing information to achieve advantages and (ii) the interviewee safeguarding against costs of disclosure. Accordingly, interviewees primarily consider the potential outcomes of disclosure when deciding what information could be shared. We can think of such possible outcomes in terms of their valence: the extent to which the expected outcome is *beneficial or costly to the interviewee*. Improving community safety by sharing useful information about a criminal gang is an example of a possible beneficial outcome. Conversely, reprisal from the criminal gang is an example of a potentially costly outcome. An interviewee may want to avoid reprisal by withholding information that incriminates the gang.

Interviewees pursue their self-interests by instrumentally disclosing information that will foster the likelihood of their overarching goal. In that regard, we posit an interplay whereby interviewees implicitly or explicitly compare the potential outcomes of disclosing to their overarching goal. They then estimate the extent to which disclosure will facilitate or impede the goal [16,18]. Therefore, an interviewee’s likelihood to disclose an information unit inherently derives from their self-interest dilemma.

**Figure 1.**
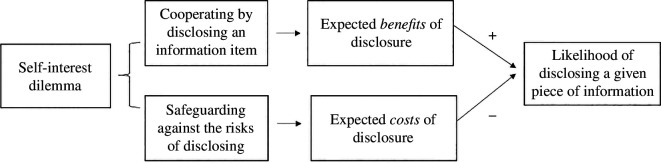
Overview of the disclosure-outcomes management model. Note: the positive sign indicates an inclination to disclosure and the negative sign indicates an inclination to non-disclosure. This depiction is not to suggest that the interviewee aggregates the expected utilities of disclosure and non-disclosure into a single expected utility. Rather, a comparison of the utilities determines likelihood that the interviewee will utter the information item.

Figure 1 illustrates how the valence of expected outcomes influences the likelihood of disclosing information. The illustration depicts the interviewees’ typical self-interest dilemma involving conflicting goals: the motive to cooperate by sharing an information item while safeguarding against the corresponding risks. The desirable outcome or benefit an interviewee expects from a decision to disclose enhances the interviewee’s likelihood to disclose. The undesirable or costly outcome the interviewee anticipates might emerge from that decision inclines them towards non-disclosure of the information in question.

Our model captures the influence exerted by the expected utilities of disclosure and non-disclosure. The interviewee compares the perceived benefits and costs of *possibly disclosing* what an interviewer is soliciting. The result of that comparison determines the likelihood that the interviewee will utter the information item in question: disclosure and non-disclosure are two sides of the same coin. When the perceived benefits exceed the costs, an interviewee will be more likely to share the information. One will be less likely to disclose when the perceived costs are higher than the benefits.

It is useful to explain how interviewees evaluate the expected outcomes of disclosing information. We have formulated such a conceptualization in qualitative terms: the *magnitude* (i.e. low and high) connected to the *valence* (i.e. costs and benefits) of an expected outcome. In this conceptualization, there are four possible combinations of expected outcomes. A given piece of information can be associated with one of those combinations—at a specific time point. These four *information types* arise from an interaction between two features of the interviewee: the characteristics of the distinct pieces of information the interviewee holds and the interviewee’s self-interest dilemma. Classifying information into one of these four categories is necessarily a caricature of variables. The classification may be more accurately represented quantitatively rather than categorically. Nevertheless, we believe the benefits of this admitted oversimplification allow enhanced comprehensibility. That advantage currently outweighs the drawback of presumed overgeneralization. Below, we elaborate on each hypothesized type of information that emerges from the described approach.

#### 1.1.1. Low-stakes information

The costs and benefits of some expected outcomes can both be low in magnitude. Such an instance could arise when there is no tangible reward to gain for sharing the information, and there is a minimal likelihood of reprisal. If disclosed, these information units have relatively few or unimportant consequences towards profitably navigating the self-interest dilemma. However, we propose that interviewees will refrain from disclosing low-stakes information to avoid taking unnecessary risks. Such disclosure is not immediately beneficial to navigating the self-interest dilemma but carries potential costs.

#### 1.1.2. Guarded information

Sometimes, the benefits of revealing a piece of information can be low, while the costs of disclosing it are high. For example, an interviewee might expect no tangible reward for sharing the information, and there is an immediate possibility of reprisal. Here, sating the interviewer’s information objectives and the interviewee’s self-interests are in direct opposition. The interviewee’s principal focus will, thus, be geared towards safeguarding their self-interest, avoiding reprisal rather than satisfying the interviewer’s information objectives. The interviewee’s navigation of the self-interest dilemma would primarily centre on protecting the information. Accordingly, interviewees will tend to be unyielding, making them unlikely to disclose *guarded information.*

#### 1.1.3. Unguarded information

An interviewee could expect disclosing an information unit to yield a highly beneficial outcome and little to no costly consequences. Such an instance could arise when one anticipates gaining an appealing reward for sharing the information and expects no reprisal in return. Disclosing this type of information ultimately serves one’s self-interests. Here, the interviewee will likely navigate the self-interest dilemma in a way that considerably satisfies the interviewer’s information objectives. Consequently, interviewees will be maximally likely to assist the interviewer by disclosing *unguarded information* to achieve the desired benefit. Interviewees may volunteer unguarded information, possibly without much or any prompting by an interviewer.

#### 1.1.4. High-stakes information

The costs and benefits of some expected outcomes can be both high in magnitude. For example, an interviewee might be anticipating gaining a tangible reward for sharing the information, but there is an immediate possibility of reprisal. These situations elicit a stark conflict between the motives of satisfying the interviewer’s objectives and safeguarding one’s interests. Both motives are important, and the interviewee cannot achieve one without seriously damaging the pursuit of the other. The strength of the conflict will likely compel the interviewee to navigate the self-interest dilemma in a way that strongly favours either enacting or evading disclosure [19]. Thus, *high-stakes information* is characterized by a highly variable likelihood of being disclosed.

## 2. Preliminary study

We conducted a preliminary study for two reasons. To provide an initial test of the procedures purposed to examine the theoretical ideas described above; and to generate data to refine our quantitative predictions. We pre-registered the planned sample size and procedures without an analysis plan: https://osf.io/dksqc?view_only=04c8b35d17b1481785729a412165f0ab.

### 2.1. Method

#### 2.1.1. Participants

We recruited participants (age ≥ 18 years) via a university participant pool and email advertisements. A total of *n* = 409 people clicked the link inviting them to participate. We excluded 109 because they failed at least one memory check or did not complete substantial portions of the experiment. The age range in the final sample (*n* = 300) was 21–64 years (*M* = 31.6, s.d. = 7.1, median = 30, missing = 41). They were 67.7% female (*n* = 203), 24.7% male (*n* = 74), and 7.6% preferred not to say (*n* = 23). Participants provided informed consent to the protocol before the experiment and received a full debriefing after.

#### 2.1.2. Procedure and materials

The procedure was entirely online, and we report only the critical aspects of the protocol in the interests of concision. Later, we provide a link allowing the reader to review this preliminary study as participants experienced it. The present procedure is highly similar to the replication study, which we will report in exhaustive detail. The research adheres to the ethical guidelines of the Swedish Research Council and applicable laws. Participants provided informed consent to the protocol before the experiment and received a debriefing after.

An incentive-compatible procedure allowed us to examine how perceived costs and benefits affect disclosure. The protocol manipulated the points one could earn based on their information disclosure. The instructions told participants that we would compile a leaderboard wherein the top five participants would win approximately 106, 84, 63, 42 and 21 USD, respectively, at the end of the research. As described below, participants received estimates of the potential costs and benefits disclosing information units could attract. However, they did not know, for sure, which information items would be costly or beneficial to disclose. Participants knew that disclosing costly information would detract points and that beneficial information would earn points. This incentive-compatible structure made the self-interest dilemma and potential disclosure outcomes tangible, aiming to mirror consequential intelligence interviews. Participants had to carefully consider the information to disclose to top the leaderboard and gain the winnings. Indiscriminate behaviour left one susceptible to losing the winnings. After data collection, the respective winners received the prizes.

##### 2.1.2.1. The source role

Participants read a background story and instructions to assume the role of an intelligence source. These instructions included conflicting motivations between cooperating with the interviewer investigating a criminal gang (i.e. to assist with their apprehension) and safeguarding oneself from risks of the gang’s reprisal. These positive and negative outcomes were represented using a point system described subsequently. Participants received a briefing about the point system in the general instructions.

##### 2.1.2.2. Information disclosure decisions

After receiving the general instructions and background, participants watched a video to better immerse them in the role. An actor portrayed an investigator who explained they were interested in information about the gang described in the background materials.

Participants then read a series of four scenarios presented in random order. We framed each scenario within the broader context of the investigation of the gang. For each scenario, participants made decisions to disclose or not disclose six pieces of information to the investigators. We presented the information pieces in a list, which was randomized per participant. Each piece of information came with a brief narrative description (e.g. the gangsters meet in the woods) and two probabilities: the probability of a positive outcome (presented as ‘XX% safe’) and the probability of a negative outcome (‘XX% dangerous’). Disclosing a given piece of information would incur an outcome based on a random process—but based on the provided probabilities. Positive outcomes provided the participant with two points, and negative outcomes detracted two points. If the sum of the probabilities of positive and negative outcomes did not sum to 100%, the remainder represented neither a positive nor a negative outcome. After each scenario, participants were given an automated update on their new current point total, providing them with feedback on the outcomes of their decisions.

The probability of a negative outcome represents the potential costs of disclosure, and the probability of a positive outcome represents its benefits. We manipulated these probabilities to create the four information types: unguarded (50% safe, 15% dangerous), guarded (15% safe, 50% dangerous), low-stakes (15% safe, 15% dangerous) and high-stakes (50% safe, 50% dangerous). The six pieces of information in each scenario were a mix of the information types. The composition of each information type differed across each scenario, but each information type was presented six times across the four scenarios. With four scenarios, each involving six disclosure decisions, each participant provided a total of 24 decisions. With *n* = 300 participants, we had a total of 7200 observations.

A snapshot of the preliminary study (animated image) is available here: https://osf.io/vxhtj.

### 2.2. Results and discussion

#### 2.2.1. Visualization

**Figure 2.**
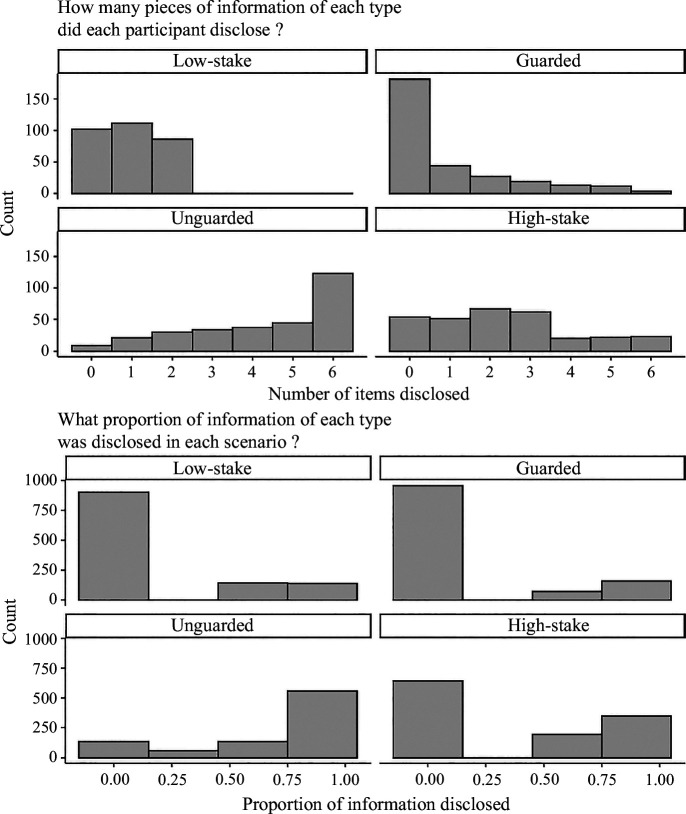
Information disclosure in the preliminary study.

As an initial exploration into the effects of the costs and benefits of disclosure on decision-making, we can examine two visualizations of participants’ disclosures (see figure 2). First, we can examine the number of pieces of each information type disclosed by each participant (displayed in the top panel of figure 2). One can see that these frequency distributions clearly demonstrate different shapes for each information type. Simple visual inspection indicates that the varying levels of costs and benefits combine to produce distinctive patterns of responses across participants.

Second, we can examine the proportion of disclosures for each type of information in each scenario (up to four proportions per participant per information type; displayed in the bottom panel of figure 2). Here, one can see several distributions that are bimodal, with values concentrated at the extremes of the scale. However, the study’s design may have exaggerated the bimodality of these distributions. Since some of the scenarios included only a single item from an information type, in those cases, the only valid values were 0 and 1. That being said, these distributions are highly similar across each of the four scenarios (see https://osf.io/nyz9s). Moreover, the distribution of high-stakes information appears to have the most pronounced bimodality. This bimodal distribution is consistent with the hypothesis that high-stakes information leads people towards highly forthcoming or highly withholding approaches, within individuals, at any given decision point (i. scenario).^1^

When trying to make sense of these data distributions, consider the non-independence of the disclosure of information units within people. Previous research has rarely explicitly addressed individual differences in the propensity to disclose information or in the sensitivity to costs and benefits of disclosure. The past research that provides data on this issue, as well as the present preliminary study, suggests that there are substantial individual differences, such that people’s decisions to disclose one piece of information are correlated with their decision to disclose other pieces [20]. Considering this correlated structure of hypothetical data leads to useful insights concerning the distributions of disclosed information. If interviewees’ disclosure decisions are substantially correlated, at any given time, across interviewees, the distribution of the proportions of disclosed high-stakes information may be bimodal or multimodal.

To understand how this finding could be the case, consider a hypothetical binomial data generation process in which 100 people are each flipping an unfair coin 100 times. Each person’s coin is unfair to different degrees, and the outcome of each person’s 100 coin flips will be correlated (i.e. the coin landing on tails is predictive of that person’s other flips also being tails). If the intra-individual correlations are substantial, the distribution of proportions (or frequencies) of successes may be bimodal (or multimodal, depending on the distribution of unfairness in the coins), since those with coins weighted heavily towards heads will accrue many successes, and those with coins weighted heavily towards tails will accrue many failures.

Following the same principles, substantial intra-individual correlations of information disclosure decisions may lead to bimodal or multimodal distributions of the proportion of disclosed information. Indeed, previous research on interviewing, including the present preliminary study, has repeatedly observed bimodal distributions of information disclosure [10,12,17,20]. However, previous research has not typically used the designs necessary to estimate these within-individual correlations. The present research has a design that addresses this issue (i.e. repeated measures of information disclosure). These correlations can be estimated as random effects in a mixed-effects model. In the present conceptualization, correlations between interviewees’ responses can be represented by random intercepts and slopes, reflecting a baseline propensity to disclose information (intercepts) and sensitivities to perceived costs and benefits (slopes). We turn to such an analysis now.

#### 2.2.2. Logistic regression modelling

To examine the influence of costs and benefits on decisions to disclose, we tested a series of three logistic regression models:

1. A fixed-effects model in which the decision to disclose a given piece of information was predicted from its costs (treatment coded: 0 = low costs, 1 = high costs) and benefits (treatment coded: 0 = low benefits, 1 = high benefit). This model assumed that all observations (i.e. each disclosure decision) were independent, despite the nested structure of the data in which multiple decisions were made by the same people in four scenarios.
2. A fixed-effects model in which we added an interaction term for costs and benefits.
3. A mixed-effects model in which we added random intercepts for each participant and each unique information item. Additionally, we modelled random slopes for each participant, for costs, benefits, and their interaction.

We compared these models using likelihood ratio tests and by examining Akaike information criterion (AIC) values. Each model outperformed the previous: Model 1 (AIC = 7857.72) versus Model 2 (AIC = 7706.74), *χ^2^* (1) = 152.98, *p* < 0.001 and Model 2 versus Model 3 (AIC = 5993.10), *χ^2^* (11) = 1735.64, *p* < 0.001. Thus, we retained the mixed-effects model for interpretation, Nakagawa *R* ^2^ (conditional) = 0.79.

Guarded information was not disclosed at significantly higher rates than low-stakes information (i.e. the coefficient for cost is non-significant). However, benefits and the interaction of costs and benefits were significant predictors of the decision to disclose information. Unguarded information was disclosed at significantly higher rates than low-stakes information. High-stakes information was also disclosed at higher rates than low-stakes information, but not to the same extent as unguarded information.

The substantial improvement in model fit from the addition of random effects suggests the presence of meaningful individual differences in the propensity to disclose information and the sensitivity to the costs and benefits of disclosure. Indeed, as can be seen in table 1, the random effect variance for both intercepts and slopes is considerable. In addition to the variance associated with individual people, different items of information vary in their likelihood of being disclosed. In an unconditional model predicting disclosure using only random intercepts for participants and for items, there was considerable variance associated with participants, ICC = 0.101, and with items of information, ICC = 0.655. A model featuring fixed effects for costs and benefits, there was also considerable variance associated with participants, ICC = 0.150, and with items of information, ICC = 0.486. These results suggest that extraneous factors exerted substantial influence on people’s decisions (e.g. the narrative content of the item, rather than the numerical cost and benefit).

**Table 1.** Fixed and random effects of costs, benefits and their interaction.

fixed effects		estimate [95% CI]
intercept		−4.63 [−3.04, −6.23]
costs		1.46 [3.59, −0.67]
benefits		6.33 [8.45, 4.21]
costs × benefits		−3.76 [−0.88, −6.64]
random effects		variance
participants	intercepts	0.892
	costs	4.141
	benefits	3.454
	costs × benefits	2.912
items	intercepts	2.923

The preliminary study’s data and analysis code are available here: https://osf.io/5rbu6/?view_only=1db497ff4e7c4f6cb9d2aeb7c5b177c7.

## 3. Replication study: the present research

The preliminary study was to determine design feasibility, finetune our hypotheses and work out an analysis plan for thorough pre-registration. This follow-up study served as a conceptual replication. Additionally, the follow-up aimed to provide insight into the credibility of the findings thus far. The research design was similar to the preliminary study. We maintained the previous instructions telling participants that we would compile a leaderboard. At the end of the research, the top five participants would win the equivalent of 106, 84, 63, 42 and 21 USD, respectively. However, the present study included variations to improve the research design and examine the replicability of our predictions.

In the preliminary study, participants’ decisions yielded multiple outcomes. They lost points, gained points or there was no change. However, the primary feedback that recurred throughout the experiment displayed total points only, merging all the consequences of decisions. In more naturalistic contexts, sources can simultaneously experience desirable, undesirable and neutral outcomes. For example, revealing to investigators the hideout of a criminal gang might both advance your goals (e.g. assisting in promoting community safety) and have damaging effects (e.g. contributing to potential retaliation against you).

We modified the structure of the replication study to allow participants to experience the potential positive, negative and neutral outcomes simultaneously. The primary feedback recorded benefits and costs separately and displayed them as participants engaged with the experiment. However, we combined those separate values at the end of the study to determine the participant’s overall performance—their points for the leaderboard. We believe these modifications made the multiplicity of outcomes salient.

The pairing of cost–benefit probabilities in the preliminary study was completely random, not minding the background story’s plot. The findings indicated that extraneous factors, likely the narrative content, may have influenced decisions. Drawing on the story’s plot, some participants may have construed their own estimates of danger and benefit probabilities, apart from the numerical manipulations. Additionally, the composition of information types differed across each scenario. That aspect may have also contributed to participants construing some scenarios as more or less dangerous than others or more or less beneficial to the ongoing investigation.

We modified the structure of the background story and the scenarios to prevent the limitations just described. In the current design, the plot indicated information items that might be more or less beneficial or dangerous to disclose. And those suggestions aligned with the numerical cost–benefit manipulations, making the potentially dangerous and beneficial disclosures arguably plausible. Additionally, the scenarios in the background story each contained an equal amount of the respective information types.

Another limitation of the preliminary study was the framing of the instructions. Generally, we told participants that they *would* earn or lose points if they disclosed beneficial or dangerous information. In truth, any of those disclosures could yield any of the possible outcomes despite the information’s description. The replication study’s instructions better reflected that the consequences of disclosures would be uncertain. We told participants that beneficial or dangerous disclosures *could* earn or detract points (see appendix).

### 3.1. Method

We pre-registered the hypotheses, power analyses, procedures, materials, data exclusion criteria, and analysis plan before data collection. The pre-registration is available here: https://osf.io/ru8j5.

#### 3.1.1. Participants

We aimed to maintain a minimum sample size of *n* = 300 participants (age ≥ 18 years) after data exclusions. Prospective participants (students and community members) were notified of the study via a university participant pool and online advertisements. The preliminary study indicated that *n* = 300 was sufficient to detect the effect sizes of interest (see §3.2); moreover, that sample size was feasible given the resources available to us.

A total of *n* = 521 began the study, and of those, *n* = 153 were excluded for not correctly responding to the attention checks. The age range in the final sample (*n* = 369) was 20–87 years (*M* = 33.20, s.d. = 11, median = 30, missing = 43). They were 72.6% female (*n* = 246), 24.8% male (*n* = 84), and 2.7% preferred not to say (*n* = 9).

#### 3.1.2. Procedure

The procedure was entirely online, like the preliminary study. Participants provided informed consent before the research commenced and received a full debriefing when we completed the research.

##### 3.1.2.1. The source role

The study was introduced as an ongoing investigation of a fictitious criminal gang. Participants read a background story to assume the role of an intelligence source who has discovered various pieces of information that may be useful to the investigation. Intelligence-gathering research often employs background stories to create source roles [21].

The background story mimics the typical intelligence scenario by including a self-interest dilemma. The source role included conflicting motivations between cooperating with an interviewer investigating a criminal gang and safeguarding oneself from the risks of the gang’s reprisal. Providing information to the interviewer may attract reprisal from the gang. However, we included a desirable outcome. Sharing information may assist the interviewer in apprehending the gang members who pose a threat to a close friend’s nephew. The gang is presumably threatening the boy to peddle drugs on their behalf at a high school. The close friend brokered an arrangement whereby the source might voluntarily provide information on the gang to the interviewer. The arrangement is ostensibly feasible because the source works at a café overlooking a park where the gang operates. Hence, the source can gather potentially useful information.

We included two memory checks to flag and exclude the data of inattentive participants who fail both or one of the checks. Appendix A contains the background story and the corresponding memory checks.

##### 3.1.2.2. Potential disclosure outcomes

After reading the background story, participants were introduced to the potential outcomes of disclosure. Similar to the preliminary study, we used an incentive-compatible points system (see [22,23] on incentive compatibility). The instructions specified the extent to which disclosing an information unit is likely beneficial and costly to the source role. Participants were told that each unit of information to be later considered for disclosure (e.g. ‘the gang comprises 10 members’) would come with two probabilities: the probability of a positive outcome (presented as ‘XX% beneficial’) and the probability of a negative outcome (‘XX% dangerous’). Sharing a given information unit incurs weighted random outcomes, positive and negative, based on the provided probabilities.

Positive outcomes would reward participants with two ‘investigation’ points, which were said to represent the extent to which the investigation is successfully proceeding against the gang. Negative outcomes would accrue two ‘danger’ points, representing dangerous disclosures that would contribute to attracting the gang’s retaliation. Thus, the probability of a negative outcome represented the potential costs of disclosure, and the probability of a positive outcome represented its benefits. We manipulated these probabilities to create the information types: unguarded (50% beneficial, 15% dangerous), guarded (15% beneficial, 50% dangerous), low-stakes (15% beneficial, 15% dangerous) and high-stakes (50% beneficial, 50% dangerous).

Our goal was to model the typical intelligence context in which sources could choose to gamble with the costs and benefits of silence. That aspect of the instructions was twofold. We informed participants that silence in any given scenario could be beneficial. Providing the investigators with no information means the source does not risk potential retaliation by the gang. However, silence allows the gang to thrive, which means the boy will remain in an unpredictable level of danger. As such, silence may lead sources to lose or gain a random number of points.

The *beneficial* information, which was safe to disclose, and the costly information, which was *dangerous* to disclose, was uncertain. The information-type manipulations indicated the potential outcomes of disclosure, but participants could not determine beyond any doubt which disclosures would actually incur benefits or costs. Hence, there would be no way to exploit the process. That protocol made it clear that the most prudent way to behave was to indicate one’s true preferences to take ownership of the decision outcomes. Random responses could not guarantee success or allay the risks.

After the instructions on potential disclosure outcomes, participants answered memory checks to assist us in flagging and excluding inattentive respondents. Appendix B contains the instructions for the points system and the corresponding memory checks.

##### 3.1.2.3. Meeting the interviewer

After the introduction to the points system, the sources meet the interviewer via a video recording (see appendix C). We recorded the video using a first-person perspective. The interviewer talked to the camera as if speaking directly to the viewer. The video allowed us to enhance this online research’s realism by varying the stimuli formats, keeping participants engaged. The interviewer introduced herself, thanked the source for taking the meeting and mentioned that she is interested in any information the source discovers about the gang. Finally, she indicated that the source was not obligated to provide any information. After the interviewer’s briefing, the instructions reiterated the potential outcomes of sharing information with the interviewer.

##### 3.1.2.4. Disclosure decisions

After meeting the interviewer, participants read three separate scenarios, each framed within the broader context of the background story. The scenarios were presented in random order. For each scenario, participants decided to disclose or not disclose 16 pieces of information to the interviewer. Those 16 units were an equal mix of the information types. Hence, each information type was presented 12 times in total.

As mentioned, we presented each piece of information with a brief description and two probabilities: for example, *the gang comprises 10 members [15% beneficial, 15% dangerous]*. The instructions explicitly mentioned to participants that they were free to choose more than one item or select none of the items if they wish to be silent. After each scenario, participants received an automated update on their current points (positive and negative). That update was designed to give them feedback on the outcomes of their decisions. The *possibility* of earning investigation points or incurring danger points aligned with the previously described probabilities corresponding to the respective information types.

*Unguarded information* (50% beneficial, 15% dangerous) included six items that earned investigation points, two items incurred danger points and four items will have no effect.

*Guarded information* (15% beneficial, 50% dangerous) included two information units that earned investigation points, six units incurred danger points and four guarded units have no effect.

*High-stakes information* (50% beneficial, 50% dangerous) comprised six units that earned investigation points and six items incurred danger points.

*Low-stakes information* (15% beneficial, 15% dangerous) comprised two items that earned investigation points, two items incurred danger points and eight low-stakes items have no effect.

The narrative for each scenario came with a list containing the information items to be considered for disclosure. We randomized the order of the list per participant. Additionally, we randomly designated the specific information units that earned investigation points or incurred danger points. We also used three different randomizations as extra safeguards to eliminate potential item and order effects and to prevent participants from possibly exploiting the process. Appendix D contains the scenarios and the code we used to randomize decisions that earned or detracted points. With three scenarios and 16 disclosure decisions per scenario, each participant will provide 48 decisions. With *n* = 300 participants, we estimate to collect 14 400 observations.

Table 2 provides an overview of the research design, and table 3 is a study design template. The preliminary study’s results indicate that the current procedure protocol is comprehensible to participants. A snapshot of the replication study (animated image) is available here: https://osf.io/shrac.

**Table 2.** Overview of the research design.

phase 1	phase 2	phase 3	phase 4	phase 5
background story introducing source role and self-interest dilemma	introduction of potential disclosure outcomes via the points system	via a video recording, the interviewer introduces herself and indicates she is interested in anything the source discovers	reiteration of the potential outcomes of disclosure in light of the self-interest dilemma	participants make their disclosure decisions across three scenarios. Each information type will be presented 12 times across the three scenarios

**Table 3.** Study design template.

question	hypotheses	sampling plan and test sensitivity rationale	analysis plan	theory that could be shown wrong by outcomes
to what extent do self-interest dilemmas generate the information types the disclosure-outcomes management model predicts?	*low-stakes information*: interviewees will refrain from disclosing low-stakes information. *Guarded information*: interviewees will be unyieldingly unwilling to disclose guarded information. *Unguarded information*: interviewees will be maximally willing to disclose things that have the features of unguarded information. *High-stakes information*: interviewees are likely to either disclose or withhold the information entirely. These four predictions are interconnected and will be tested by the benefit coefficient and the interaction term	we aimed to include a minimum of *n* = 300 participants. Each participant will make 48 decisions, which will provide an approximate total of 14 400 observations in the present study. See §3.2 for power calculations	a series of mixed-effects logistic regression models (significance threshold = 0.05). The model selection will take an additive approach, wherein fixed and random effects are added in progressive steps. The risk and benefit effects and their interaction will provide information about whether the predictions (i.e. information types) bear out here. To support the hypotheses, the coefficient for benefit should be positive, and the interaction should be negative	due to power considerations, the disclosure-outcomes management model cannot necessarily be disproven here. The replication study does not have adequate power to detect effects that are substantially smaller than those observed in the preliminary study. Because of that limitation, if the results are non-significant, we cannot make claims about the absence of theoretically relevant effects. This research will assist in determining whether the model’s tenets are worth pursuing in future research

### 3.2. Analysis plan

We tested our hypotheses using mixed-effects logistic regression modelling. We fit a series of models predicting participants’ decisions to disclose (or not to disclose) each piece of information. We fit the following models:

1. A model with the information’s costs (0 = low, 1 = high) and benefits (0 = low, 1= high) predicting the decision to disclose (0 = did not disclose, 1 = disclosed), with random intercepts for each participant and each information item as well as random slopes for each participant for costs and benefits.
2. A model adding the interaction of costs and benefits as a fixed effect, as well as random slopes for this interaction for each participant.

Models were compared using likelihood ratio tests (significance threshold = 0.05). We retained for interpretation the model that best fitted the data according to these tests. All examined models are documented and reported either in the main text or supplementary material. Models were fitted using the *lme4* package [24] for R (R Core Team, 2022). Model convergence was evaluated using the *glmer()* function’s defaults, but planned to override the defaults to specify that the optimizer will perform 100 000 function evaluations at maximum. If a model failed to converge, we planned to remove it from consideration for retention and interpretation.

The primary effects of interest were the fixed effects for risk and benefit and the random effects for individual participants. The risk and benefit effects provided information about the extent of support for the disclosure-outcomes management model. To support the hypotheses, consistent with the preliminary study, the coefficient for benefit should be positive, and the interaction should be negative.

To assess statistical power, we conducted a simulation-based power analysis using the *simr* package for R [25]. The analysis drew on the preliminary study’s results: coefficient for benefits, *b* = 6.33, 95% CI [4.21, 8.45] and a negative interaction between risk and benefits, *b* = −3.76, 95% CI [−6.64, −0.88]. Using our planned sample size of *n* = 300 participants and using the fixed effects and random effects variances observed in the preliminary study, we examined statistical power for the interaction between risks and benefits under three conditions: (i) with the same effect observed in the preliminary study; (ii) with an effect half the size as the previously observed effect; and (iii) with an effect equal to the bound of the 95% CI of the original effect that was closer to 0.

Under those three assumptions, we found that this sample size will respectively provide 95% power for *b* = −3.76, 46% power for *b* = −1.88 and 16% power for *b* = −0.88. The replication study had considerable power to detect effects similar in size to the previously observed effects. However, the replication study does not have adequate power to detect effects that are substantially smaller. Because of this limitation, we cannot make claims about the absence of theoretically relevant effects—for statistically non-significant results. One can access the power analysis here: https://osf.io/5rbu6/.

### 3.3. Results and discussion

**Figure 3.**
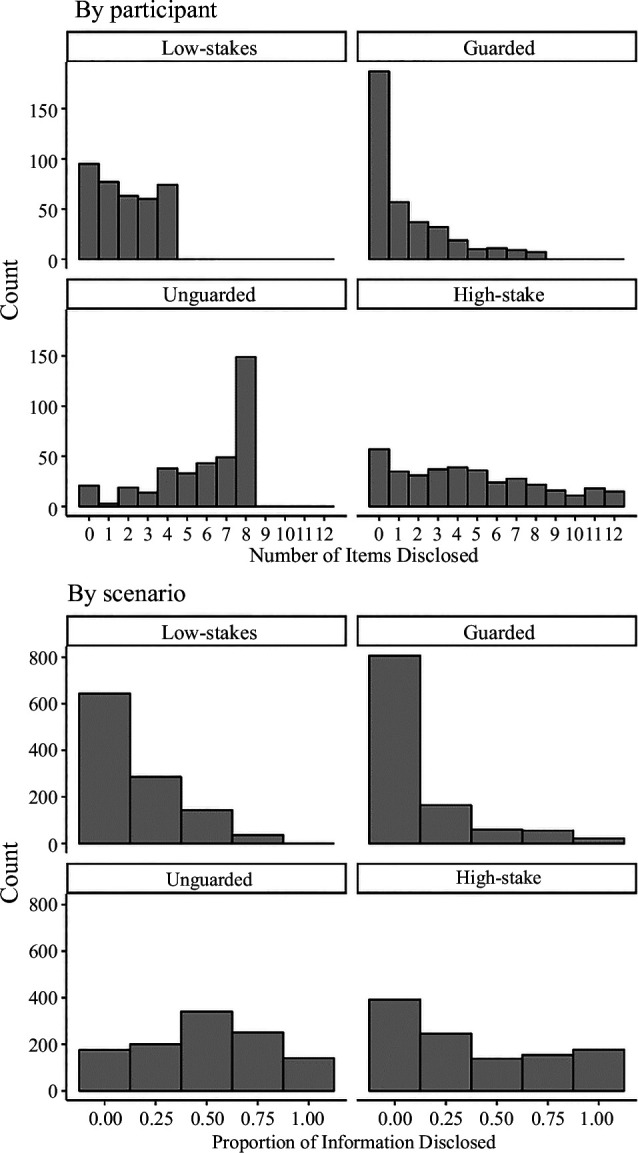
Information disclosure in the replication study. Note: there were 12 items in each information category and 3 scenarios.

With a total sample size of *n* = 369 participants, each making 48 decisions about information items, the data included a total of 17 712 disclosure decisions. Across scenarios, participants disclosed 49.5% of the unguarded information, 12.1% of the guarded information, 15.3% of the low-stakes information and 38.2% of the high-stakes information. These findings broadly align with our predictions. The frequency distributions of information disclosure by participant and by scenario are displayed in figure 3.

The logistic regression model, including the interaction between costs and benefits, fits the data better than the model without the interaction, *χ* ^2^(5) = 26.29, *p* < 0.001, Nakagawa *R* ^2^ (conditional) = 0.662, AIC 14 969 versus 14 952. The results of this model are displayed in table 4. As predicted, the coefficient for benefits was positive, indicating that participants were more likely to disclose unguarded and high-stakes information compared with low-stakes information. However, against predictions and failing to replicate the preliminary study, the interaction coefficient was non-significant. In an unconditional model, we found that there was substantial random effects variance associated with participants, ICC = 0.12, and with information items, ICC = 55. We additionally examined the variance in a model adding costs and benefits as fixed effects, which also demonstrated substantial variance for participants, ICC = 0.15, and for items, ICC = 0.43.

**Table 4.** Mixed-effects logistic regression model.

fixed				
coefficient	*b* (log odds)	95% CI	*z*	*p*
intercept	−2.63	[−3.78, −1.49]	4.51	<0.001
cost	−0.42	[−2.03, 1.19]	0.51	0.61
benefit	2.15	[0.55, 3.75]	2.64	0.008
cost × benefit	0.21	[−2.04, 2.45]	0.18	0.86
random				
group	term	s.d.		
participants	intercepts	1.06		
	cost	1.36		
	benefit	0.78		
	cost × benefit	0.69		
item	intercepts	1.69		

In the planned conditional model, there was a considerable amount of variance in the random slopes across participants for costs, benefits and their interaction. Thus, the results did not support the prediction concerning the interaction of costs and benefits; instead, they indicate that people vary substantially in their sensitivity to costs and to the combination of high costs and benefits.

## 4. General discussion

We theorized about the mechanisms by which intelligence interviewees (or sources) decide what to disclose. A core tenet of our proposal was that the goals an interviewee can accomplish by disclosing and withholding the same information are often in tension—that is to say, disclosure and non-disclosure are two sides of the same coin. Thus, interviewees engage in an *intuitive* cost–benefit analysis when deciding what could be disclosed. The findings were broadly in line with our proposition that interviewees consider the potential outcomes of their disclosures and are likelier to disclose information items they believe would maximize perceived benefits and incur the least costs.

The findings from the preliminary and replication studies indicated that perceived benefits positively influenced the likelihood of disclosing. The effect of the benefits of disclosure was statistically significant across the studies. Unguarded and high-stakes information were the information items that carried a higher likelihood of benefits, and interviewees disclosed those information items at higher rates than low-stakes and guarded information. Furthermore, the effect of the cost of disclosure was statistically significant in neither of the studies. Interviewees were not less likely to disclose guarded information compared with low-stakes information. In our research design, guarded information items came with greater risks than low-stakes information but not greater benefits.

However, the negative interaction between costs and benefits observed in the Preliminary Study did not replicate in the Replication Study. That finding may be due to power constraints, not evidence against the existence of an interaction effect. We did not power the replication to determine the absence of an interaction effect. Our proposal that—generally speaking—interviewees are likelier to disclose information units that seem less versus more risky requires further examination. Our manipulation of costs and benefits did not exert a consistent influence across participants and across the studies. In the Replication Study, the logistic regression model, including the interaction of costs and benefits, fitted the data better primarily because of the random effects variance of the interaction effect. That is, the average interaction effect was close to 0, but the variance suggests that interviewees seem to vary substantially in their sensitivity to high costs and benefits when deciding what to disclose [26]. People vary substantially in how they handle high-stakes information. We do not yet have indisputable grounds to claim there was or was not an interaction between costs and benefits on average, though we believe there is evidence that people vary widely in their sensitivity to benefits, costs and their co-occurrence. Our best speculation, at this time, is that our efforts to control variance stemming from the background story’s narrative content did not work. Participants in the Replication Study, like in the Preliminary Study, may have construed their own estimates of cost and benefit probabilities based on the narrative content (as well as other idiosyncratic considerations), not minding the numerical manipulations. We can think of two possibilities for future research, given the volatility of individual risk appetite regarding disclosure decisions.

Further studies could explore factors that invite uncertainty about perceived costs and benefits. A relevant variable here is discounting—the influence of outcome proximity. Future studies might examine how outcome proximity might affect interviewees’ reactions to high costs and benefits. The outcomes of disclosure decisions in the present research were immediate; participants experienced the consequences of their decisions right after making them. We cannot definitively say whether or how that design choice influenced disclosure compared to a comparison condition with delayed consequences. Some participants may have construed, based on the background story, that the gang’s retaliation (i.e. costs) or interdiction (i.e. benefits) might come sooner or later—not minding the points system in the respective studies. We did not plan to actively manipulate discounting; in fact, our theoretical propositions do not yet include hypotheses on discounting. Nonetheless, the broader decision-making literature indicates that people give more credence to proximal outcomes: they crave immediate benefits than later ones and are more likely to defer costs if they can [27]. Actively manipulating outcome proximity might provide more insights into how people manage disclosure in intelligence interviews.

Another opportunity for future work is to embrace the possibility of uncontrollable variance in interviewees’ sensitivities to costs and benefits regarding disclosure. Even if we designed the perfect experiment that completely controlled cost–benefit sensitivities, such a design might suffer from ecological validity limitations that cannot be ignored. If interviewees’ idiosyncrasies could not be constrained in the present tightly controlled studies, then those peculiarities are likely to manifest in the field where minimal control can be exerted. Research that follows this path should be cognizant of individual-level variance and treat it as an active independent variable. Such work could also explore how interviewing methods might affect predictors of interviewees’ sensitivities (e.g. discounting).

### 4.1. Constraints on generality: internal and external validity

Investigative interviews usually involve verbal interactions where interviewees self-generate the information items to disclose. In such verbal exchanges, interviewees can provide irrelevant information, lie or forget about details they would have otherwise disclosed had they remembered. Another limitation is that this research cannot capture the unspoken influences of real-time conversations. For example, an interviewer might express disbelief with a frown or approval with a smile. Those expressions might affect the interviewee’s cost–benefit considerations regarding current and prospective disclosures, and an interviewee could strategically offer tentative disclosures by nodding in agreement or shaking their head to disconfirm a claim.

We acknowledge that our research is limited regarding the additional layers of interaction a verbal interview can bring. However, our current focus is to examine the mechanisms underlying what interviewees actively *choose* to disclose. The present research design allowed participants to actively choose what to disclose and is a prudent design, given our objective. The current research study cannot generalize to passive disclosures. Nonetheless, our procedure included nuance. Participants received the probabilities of disclosure outcomes before disclosure and the consequences of decisions afterward. These aspects of our design aimed to mimic the appraisal of potential outcomes, such as the perceived positive and negative interviewer reactions. Our protocols, namely the substantial prizes, made the consequences of participants’ decisions tangible, not merely imagined. Nonetheless, we must admit that the possibility of not winning a few hundred USD pales in comparison to the costs interviewees might face. This limitation with external validity is an issue that investigative interviewing research needs to surmount with creative research designs.

Studies that have used verbal interviews also include background stories. Those stories guide the coding of verbal interviews by providing predefined criteria of what constitutes legitimate disclosures as opposed to irrelevant ones and lies. Coding verbal interviews can generate new information items that researchers did not anticipate, and we acknowledge that advantage. However, our current goal was to examine the mechanisms of what interviewees choose to disclose, not the generation of new information from background stories. Additionally, coding breaks down verbal interviews into a list of legitimate items interviewees have disclosed. Our procedure retained the essential aspect of flagging legitimate disclosures and eliminated potential coding errors.

We do not intend to dismiss the psychological realism that verbal interviews can bring. We are simply defending the need to ensure internal validity. Given this early stage of examining the mechanisms of what interviewees actively choose to disclose, it was prudent to exercise maximum experimental control.

## 5. Concluding remarks

We hope this work will inspire researchers to move beyond treating disclosure as stemming from global motivation that applies equally to all the information an interviewee holds. To better understand intelligence interviewing, we must uncover why interviewees choose to disclose the specific information items they do but not others and the attendant underlying mechanisms.

## Data Availability

All data supporting the findings in this research are publicly available on the Open Science Framework repository (osf.io). This research is a Registered Report reviewed and recommended by PCI Registered Report. Recommendation links are as follows. Stage 1 recommendation and review history: [28]; Stage 2 recommendation and review history: [29].

## Footnotes

^1^
Note that the upper panel of figure 2 does not feature similar bimodality. The upper panel displays disclosure frequencies for each participant, across all scenarios they encountered, whereas the lower panel displays proportions of disclosure for participants’ decisions in each scenario. Thus, for high-stakes information, in any given scenario, participants seemed to make a decision to disclose no such information or a substantial amount of that information, producing a bimodal distribution at the scenario level. From scenario to scenario, however, a participant might have been variously highly forthcoming or highly withholding with high-stakes information, producing a different distribution at the participant level.

## Appendix A. Background story

Imagine that you are one of the workers at a big café in town. Because you have good knowledge about coffee, your duties involve making the coffee drinks customers order. You usually work from 11.00 to 18.00. Although you are not exactly friends with your co-workers, you have a decent working relationship with most of them. Sometimes, you may have small-talk during break times.

The café overlooks a public park close to the woods. You and your colleagues have a good picture of what goes on in the park. It is well known among workers at the café and around town that a gang called SK14 operates there, but no one really discusses this issue. The reason is that SK14 may be a dangerous group with connections everywhere in the city. The gang is suspected to be involved in several drive-by shootings in town, but this is an unconfirmed rumour. What is well known is that the SK14 network carries out various criminal activities. You know that one of SK14’s operations is selling narcotics in the park. You have overheard some of your colleagues talk about a special brand of *strong ecstasy* that only SK14 sells.

You have a good friend called Alvi, and you go on bike rides together some weekends. Recently, Alvi informed you that SK14 gangsters have been threatening his nephew, a 15-year-old boy. The gang wants the boy to sell their strong ecstasy at his high school. On a few occasions, some SK14 members have confronted Alvi’s nephew on his way home from soccer practice. It seems the gangsters always know where to find the boy. The police have opened an investigation into this case. However, the police have been investigating SK14 for some time before this incident.

Through Alvi, a police-contact arranged a deal with you. Your part in this deal is to continue your work at the café as usual and observe the park when you can. The plan is to meet the police-contact from time to time, and you will discuss anything you may have noticed. An important part of your deal with the police-contact is that *you are not obligated to say anything*. Whatever you decide to tell is totally up to you.

### A.1. Memory checks

Which of these tasks is included in your work at the café?

Making coffee (Pass)

Take care of electronic equipment at the café (Fail)

Which illegal drug is sold by the SK14 group?

‘Strong ecstasy’ (Pass)

Fentanyl (Fail)

## Appendix B. Points-system instructions

By participating in this experiment, you have the chance to win a cash prize. You can win 1 of 5 cash prizes depending on your total compared with the other participants in this research. Whoever completes the study with the most points achieves first place and wins 106 USD. The 2nd, 3rd, 4th and 5th place winners will win 84, 63, 42 and 21 USD, respectively. If there are ties in any of the five places, the winner of the tied position will be determined via a random draw. Your total points and chance to win any of the prizes to depend on the decisions you make in this section of the experiment. **So, please read the upcoming instructions carefully**.

When you are ready click the next button to continue: <Page break>

You will be placed in three scenarios where you will discover various bits of information about the SK14 gang. At the end of each scenario, you will indicate the bits of information you are willing to disclose to the police-contact. Depending on the situation, some bits of information will be beneficial to disclose. Such information could be potentially useful for investigating and arresting the SK14 gangsters: and this will help protect Alvi’s nephew because the gangsters will not be able to reach him. Other bits of information will be dangerous such that revealing them could attract retaliation from SK14.

— For any **beneficial** bit of information, you indicate you are willing to disclose, you could **earn** two (+2) ‘investigation points’. The more **investigation points** you earn the more likely the investigation against the gang will be successful, leading to their arrest. Then, Alvi’s cousin will be safe.
— For any **dangerous** information, you indicate you are willing to disclose, you could **incur a tax of** negative two (−2) ‘danger points’. The more **danger points** you incur, the more likely you are to attract the gang’s retaliation; you know SK14 can be dangerous.

The level of benefit and danger of disclosing each information will be marked next to the information, as in the example below.

There are 10 SK14 gangsters [15% beneficial, 15% dangerous]

When you are ready click the next button to continue: <Page break>

If you disclose a lot of beneficial information, you could earn more ‘**(+) investigation points**’. And if you disclose a lot of dangerous information, you could incur more ‘**(−) danger points**’.

If you stay silent and give the police-contact *no* information you could reduce the risk of retaliation by the gang. However, staying quiet means the gangsters might not be arrested, which means Alvi’s nephew will be in danger. Therefore, if you remain silent, you may *gain* points to protect yourself or you may *lose* points for putting Alvi’s nephew in danger.

As you can see, there is no way to exploit the process. **The beneficial and dangerous information for each scenario will be randomly determined**. You cannot be absolutely sure what information will be beneficial or dangerous to disclose. **And you do not know what will happen if you stay silent.** So, always indicate the information you are TRULY willing to disclose based on the specific events of each scenario.

When you are ready click the next button to continue: <Page break>

**Summary:** The points system is quite simple.

— Across three scenarios, you will discover bits of information about the SK14 gang. At the end of each scenario, you will decide the information you are willing to disclose to the police-contact.
— Some information will be beneficial to disclose, and others will be dangerous to disclose.
— If you disclose any beneficial information, you will earn ‘(+) investigation points’. If you disclose dangerous information, you will incur ‘(−) danger points’.
— The beneficial and dangerous information will be randomly determined. You cannot be absolutely sure what information will be beneficial or dangerous to disclose.
— Always consider the specific events in each scenario and indicate the information you are TRULY willing to disclose.

When you are ready click the next button to continue: <Page break>

### B.1. Memory checks

What happens if you share **beneficial** information?

I could earn ‘(+) investigation points’ (Pass)

I could incur ‘(−) danger points’ (Fail)

What happens if you share **dangerous** information?

I could earn ‘(+) investigation points’ (Fail)

I could incur ‘(−) danger points’ (Pass)

## Appendix C. Interviewer script

Hey, I am Kim Johansson. I am a detective here in town. Thank you for taking the time to meet with me today. As you know, we are still investigating SK14, as well as what happened to Alvi’s nephew. So, I am interested in anything you observe in the park. Please remember that you are not obligated to tell me anything. Whatever you are willing to disclose is entirely your choice.

Video: https://osf.io/fd2x4/?view_only=21f4b54d0e994ac5892edddaf4e42d66.

### C.1. Reiterating the potential outcomes of disclosure

Next, you will decide the information you are willing to disclose to the interviewer. There are potential consequences with what you decide to say.

Information items that are beneficial to disclose can contribute to arresting the SK14 gangsters: and this will help protect Alvi’s nephew because the gangsters will not be able to reach him. If a piece of information is highly beneficial to share, there will be a note indicating **50% beneficial** attached to it. Information whose benefit to disclose is low will have a note indicating **15% beneficial.**

Dangerous information items are those that could put you and your colleagues at the café in danger of SK14’s retaliation. If a piece of information is highly dangerous to share, there will be a note indicating **50% dangerous** attached to it. Information whose danger to disclose is low will have a note indicating **15% dangerous.**

The level of benefit and danger of disclosing each information will be marked next to the information, as in the example below.

There are 10 SK14 gangsters [15% beneficial, 15% dangerous]

You do not know what will happen if you stay silent: you may gain or lose points. So, always choose what you are TRULY willing to disclose based on the specific events of each scenario. When you are ready click the next button to continue: <Page break>

## Appendix D. Scenarios

Note to reviewers: the additional labels here are to facilitate the review process. The main study will include the explicit labels of the information types.

### D.1. Scenario 1

***[Low-stakes]*** One morning you decided to come to work earlier than usual for some cleaning. You arrived at approximately 08.00, but no one saw you come in. While cleaning, you noticed that the SK14 gangsters arrived in a different vehicle—a black SUV rather than their usual minivan. As you paid more attention, you saw that there were 7 of them: 3 men and 4 women. They were all dressed in outdoor sportswear, so they looked like anyone else who uses the park.

***[Unguarded]*** Later that morning, around 08.45, you saw that the gangsters were arguing. They still had not noticed you were in the café. Even though you did not hear so clearly, it seemed that they were arguing about selling a high dose of drugs to Alvi’s nephew. The gangsters are now worried that the victim’s overdose will draw attention from the police. They are thinking of a new location for the drug sales and possibly selling during early mornings only.

***[High-stakes]*** You had a long lunch break since you came to work early. So, you decided to go for a quick jog through the woods. Just before entering the wooded area, you got distracted and looked away from the path for a brief second. You bumped rather hard into a woman. Both of you were quite shocked by the event, and you paused to get a good look at each other. She was probably in her late 20s, about 165 cm tall, and had purple-dyed hair. She fell when you bumped into her, and she dropped a plastic bag spilling the contents. You noticed immediately that the contents were drugs, so you know she must be one of the SK14 gangsters. The drugs were packaged in paper pouches of about 8 cm × 8 cm. The pouches had a drawing of a horse and the number 160 under the horse.

***[Guarded]*** Just before closing time, one of the new SK14 members came into the café to buy some coffee. You know he is new because, typically, SK14 members stay away from stores in the area. The gangsters do not like their identities to be known—and they can be quite dangerous when it comes to protecting their identity. During the time the gangster placed the order, there was no milk at the counter. So, you asked your colleague to get some milk from the fridge in the back. While you were waiting, you got a good look at his face and stature. And you could tell he was observing you as well. You can guess that he is about 190 cm tall. His hair was dark with grey streaks. He had green eyes and a scar on his left jaw. The name on the card he used to pay for his drink was Kari Jupo.

#### D.1.1. Information units (Scenario 1)

**Note that the information units will be mixed and presented in random order.*

##### D.1.1.1. Low-stakes

— There were SK14 gangsters in the park around 08.00.
— There were four SK14 gangsters, in the park, who were women.
— There were three SK14 gangsters, in the park, who were men.
— The gangsters seemed to have changed their vehicle from a minivan to a black SUV.

##### D.1.1.2. Unguarded

— An argument occurred between some SK14 gangsters around 08.45.
— The gangsters argued about selling a high dose of drugs to Alvi’s nephew.
— The gangsters are worried that the recent overdose will draw attention from the police.
— The gangsters are planning to sell their drugs at a new location.

##### D.1.1.3. High-stakes

— One of the SK14 gangsters is a woman in her late 20s with purple-dyed hair.
— SK14 seems to use some kind of standard packaging for their drugs.
— SK14 labels the packaging of their drugs with a horse and the number 160.
— SK14 packages drugs in paper pouches of about 8 cm × 8 cm.

##### D.1.1.4. Guarded

— One of the SK14 gangsters is a man who is about 190 cm tall.
— One of the SK14 gangsters has green eyes and a scar on his left jaw.
— One of the SK14 gangsters has dark hair with grey streaks.
— One of the SK14 gangsters is possibly called Kari Jupo.

### D.2. Scenario 2

***[Guarded]*** You took the bus from the city centre to work. When you entered the bus two guys were sitting somewhere in the middle, and they were talking rather loudly. Perhaps, they were drunk. So, you stopped to look, and they saw you too. One of the guys pointed at you and said something to his friend. You sat two seats behind them, but you heard their conversation clearly. The guys were discussing a party they had just attended. They mentioned that the party was organized by the Owl Night Club. But was held in a private apartment close to the city centre, attendance was by invitation only. The guys supposedly got invitations to the party by frequently buying VIP tickets to the Owl Night Club. They seemed pretty excited about the fact that there was strong ecstasy at the party—you know this drug is sold only by SK14. So, SK14 must be the drug supplier for this party. The guys also mentioned that they’ve had strong ecstasy at another similar party. So, it seems that SK14 supplies to other private parties in town.

***[High-stakes]*** During your lunch break, your colleague, who is becoming friends with an SK14 member, slipped you some details. It is not clear how she got the information, but she said that SK14 is connected to a much bigger gang called TETO. TETO operates all over the country and supplies drugs to SK14 and most of the gangs in the country. TETO deals in wholesale narcotics and opioids. TETO prefers to sell to gangs but not to individuals. The police need this information because it turns out that Alvi’s nephew overdosed on an unknown drug—not oxycodone.

***[Low-stakes]*** Later that day when the café was very crowded, you noticed that the SK14 gangsters arrived at the park using off-road motorbikes instead of the SUV or minivan they typically use. They arrived around 13.00. You saw that although the gangsters had different helmets, each helmet had a thick blue line running across it. Two of the bikes had license plates labelled TROY and PECS.

***[Unguarded]*** You have noticed that there seems to be a pattern among the SK14 gangsters when they arrive at the park in the evenings. You realized their pattern when you were in the changing room preparing to go home. Usually, about 8–10 gangsters arrive at a time, just before most of the shops around close. Five of the gangsters enter the woods using the marked entrance 4B. The remaining gangsters then wait near the park benches for about 30 min, and they enter the woods using the marked entrance 7E.

#### D.2.1. Information units (Scenario 2)

**Note that the information units will be mixed and presented in random order.*

##### D.2.1.1. Guarded

— SK14 supplies drugs to parties organized by the Owl Night Club.
— SK14 may be the drug supplier for many small private parties in town.
— There was a party at a private apartment in the city centre where SK14 supplied strong ecstasy.
— To get an invitation to a party where SK14 provides drugs, one has to buy VIP tickets to the Owl Night Club.

##### D.2.1.2. High-stakes

— SK14 is connected to a much bigger gang called TETO, which operates all over the country.
— A gang called TETO supplies drugs to SK14 and most gangs in the city.
— TETO is another active gang, which deals in narcotics and opioids.
— TETO is a wholesale drug supplier to small gangs, and they do not like dealing with individuals.

##### D.2.1.3. Low-stakes

— SK14 gangsters arrived at the park at 13.00 on off-road motorbikes.
— SK14 gangsters use helmets that has a thick blue line running across it.
— SK14 gangsters use a motorbike with a license plate labelled TROY.
— SK14 gangsters use a motorbike with a license plate labelled PECS.

##### D.2.1.4. Unguarded

— SK14 typically arrives at the park in a group of 8–10 people.
— When the gangsters arrive at the park, five of them usually enter the woods first after 30 min.
— SK14 gangsters enter the woods using the marked entrance 4B.
— SK14 gangsters enter the woods using the marked entrance 7E.

### D.3. Scenario 3

***[Low-stakes]*** On your way to work, you noticed something that looked like a black wallet on one of the park benches. You decided to have a look and possibly alert the owner. When you got closer and picked up the wallet, you realized it was not a wallet. The object was a radio communication device that was blue on the front and black on the back. The brand name on the gadget was Motorola, and the serial number was MTX7782R. As soon as you put the device down, it beeped and a voice said: ‘A rabbit is on the way’. After about 20 s, the same voice said: ‘No purple carrots for…’. You did not hear the end of the sentence because you quickly put the radio down and walked away. You are not sure, but the radio could be owned by SK14.

***[Unguarded]*** While cleaning the café when you arrived at work, you noticed a green pouch of about 6 cm × 4 cm on the floor. At first, you did not pay much attention to the pouch, but as you got closer you noticed the SK14 symbol, the horse and the number 160, on the pouch: You rushed to grab it. Unfortunately, in your haste, you spilled a lot of detergent fluid on your clothes and the floor. You took a moment to gather yourself before reaching for the pouch. As you picked the pouch up, it had gone soft and tore. Two pills fell out: one was red and the other was white. Both pills had the marking GIP. Before you could grab the pills, they dissolved in the puddle of detergent they fell in.

***[High-stakes]*** During your break, you went to the nearby restaurant to get lunch. At this restaurant, one orders and pays at the counter. There was a guy right ahead of you ordering his meal. You recognized him immediately: he is an SK14 gangster. You have come across this guy in the park a few times, so he may be familiar with you as well. Previously, you never had the chance to get a good look at him; however, on this day, you did. You remember that this guy is one of the main dealers for teenagers. You recognized that he was wearing an orange sweatshirt you’ve seen him in before. He is about 175 cm tall, has brunette hair and brown eyes. There is a distinct birthmark around his right eye. He usually hangs around marked entrance 3D or 8F. You couldn’t help but pay close attention to the card he used to pay for the meal. It was not so clear, but the first name on the card was Jari.

***[Guarded]*** There is a garage called Busas close to the woods where you work. After 15.00, when Busas has closed, various motorcyclists use the Busas parking lot and they ride rather loudly. Lately, the noise has been unbearable, which is driving customers away. Usually, these motorcyclists are young teenagers, so one day, you decided to tell them to ride somewhere else. Technically, it is illegal to ride in the park after 15.00. As you approached the parking lot, you saw about six motorcyclists. You noticed these people were a bit unusual because their helmets had tinted visors. So, you could not identify who exactly was riding. When you got closer, you saw that all the bikes were grey BMW sports bikes, but there were no license plates. You noticed that one of the riders was an SK14 gangster, his jacket had a symbol of a dragon and a sword on the left breast pocket. He walked up to you and said: ‘do we have a problem? You better go back to the café!’. You said ‘of course’ and walked away.

#### D.3.1. Information units (Scenario 3)

**Note that the information units will be mixed and presented in random order.*

##### D.3.1.1. Low-stakes

— It is likely that SK14 gangsters use Motorola radio communication devices.
— A code SK14 gangsters use to communicate is possible: ‘A rabbit is on the way’.
— SK14 gangsters possibly use a radio communication device with serial number MTX7782R.
— A code SK14 gangsters use to communicate is probably: ‘No purple carrots for…’

##### D.3.1.2. Unguarded

— SK14’s drugs seem to dissolve quickly when in contact with a detergent fluid.
— SK14’s drugs have the marking GIP, and they are coloured red or white.
— The size of the green pouches SK14 uses is 6 cm × 4 cm.
— The SK14 symbol (the horse and the number 160) seems to be on every pouch containing SK14 drugs.

##### D.3.1.3. High-stakes

— One of the SK14 gangsters that deals mainly with teenagers is a male of about 175 cm with a birthmark around his right eye.
— An SK14 gangster hangs out at marked entrance 8F or 3D and deals to teenagers
— One of the SK14 gangsters is possibly called Jari.
— One of the SK14 gangsters that deals mainly with teenagers is a male who wears an orange sweatshirt and has brown eyes.

##### D.3.1.4. Guarded

— SK14 gangsters use the Busas parking lot after 15.00.
— SK14 gangsters own six grey BMW sports bikes.
— SK14 gangsters wearing helmets with tinted visors ride unlicensed motorbikes in the Busas parking lot.
— SK14 motorcyclists wear jackets with a symbol of a dragon and a sword on the left breast pocket.

### D.4. Code to randomize earnings and losses

## The results from the ‘sample’ function on each information type will be used to assign the effect of the specific pieces of information. For each information type, the assignment will be made in serial order. Consider the unguarded information type, for example; if the ‘sample’ results return ‘gain’ first in the list, then the first unguarded information item will increase the endowment, and so forth.

## The numbers attached to gain, loss and null are to ensure that the appropriate amount of gains, losses and nulls feature in each information type.

## Using the code below, we will create three different randomizations to further eliminate order effects and to prevent participants from possibly exploiting the process. We will use the ‘set.seed’ function to ensure that one can replicate the randomizations.

low.stakes ← c(‘gain.1’, ‘gain.2’, ‘loss.1’, ‘loss.2’, ‘null.1’, ‘null.2’, ‘null.3’, ‘null.4’, ‘null.5’, ‘null.6’, ‘null.7’, ‘null.8’) low.stakes.points ← sample(low.stakes) low.stakes.points unguarded ← c(‘gain.1’, ‘gain.2’, ‘gain.3’, ‘gain.4’, ‘gain.5’, ‘gain.6’, ‘loss.1’, ‘loss.2’, ‘null.1’, ‘null.2’, ‘null.3’, ‘null.4’) unguarded.points ← sample(unguarded) unguarded.points high.stakes ← c(‘gain.1’, ‘gain.2’, ‘gain.3’, ‘gain.4’, ‘gain.5’, ‘gain.6’, ‘loss.1’, ‘loss.2’, ‘loss.3’, ‘loss.4’, ‘loss.5’, ‘loss.6’) high.stakes.points ← sample(high.stakes) high.stakes.points guarded ← c(‘gain.1’, ‘gain.2’, ‘loss.1’, ‘loss.2’, ‘loss.3’, ‘loss.4’, ‘loss.5’, ‘loss.6’, ‘null.1’, ‘null.2’, ‘null.3’, ‘null.4’) guarded.points ← sample(guarded) guarded.points

# no comment gamble for each scenario no.comment.gam ← sample(−6:6)

sample(no.comment.gam, size = 3, replace = TRUE, prob = NULL)
